# Effect of Centralization on Surgical Outcome of Children Operated for Liver Tumors in Switzerland: A Retrospective Comparative Study

**DOI:** 10.3390/children9020217

**Published:** 2022-02-06

**Authors:** Jasmine Leoni, Anne-Laure Rougemont, Ana M. Calinescu, Marc Ansari, Philippe Compagnon, Jim C. H. Wilde, Barbara E. Wildhaber

**Affiliations:** 1 Swiss Pediatric Liver Center, Geneva University Hospitals, 1205 Geneva, Switzerland; Jasmine.leoni@hcuge.ch (J.L.); anne-laure.rougemont@hcuge.ch (A.-L.R.); Ana-maria.Calinescu@hcuge.ch (A.M.C.); Marc.Ansari@hcuge.ch (M.A.); Philippe.Compagnon@hcuge.ch (P.C.); Jim.wilde@hcuge.ch (J.C.H.W.); 2 Division of Child and Adolescent Surgery, Department of Women, Child and Adolescent, Geneva University Hospitals, 1205 Geneva, Switzerland; 3 Department of Pediatrics, Gynecology and Obstetrics, University of Geneva, 1205 Geneva, Switzerland; 4 Diagnostic Department, Division of Clinical Pathology, Swiss Pediatric Liver Center, Geneva University Hospitals, 1205 Geneva, Switzerland; 5 Unit of Oncology and Haematology, Department of Women, Child and Adolescent, Geneva University Hospitals and CANSEARCH Research Platform in Pediatric Oncology and Hematology, 1205 Geneva, Switzerland; 6 Department of Surgery, Division of Transplantation, University Hospitals of Geneva, 1205 Geneva, Switzerland

**Keywords:** centralization, pediatric, hepatic, surgery, oncology, complications, relapses

## Abstract

Background: Pediatric liver surgery is complex, and complications are not uncommon. Centralization of highly specialized surgery has been shown to improve quality of care. In 2012, pediatric liver surgery was centralized in Switzerland in one national center. This study analyses results before and after centralization. Methods: Retrospective monocentric comparative study. Analysis of medical records of children (0–16 years) operated for any liver tumor between 1 January 2001 and 31 December 2020. Forty-one patients were included: 14 before centralization (before 1 January 2012) and 27 after centralization (after 1 January 2012). Epidemiological, pre-, intra-, and post-operative data were collected. Fischer’s exact and *t*-test were used to compare groups. Results: The two cohorts were homogeneous. Operating time was reduced, although not significantly, from 366 to 277 min. Length of postoperative stay and mortality were not statistically different between groups. Yet, after centralization, overall postoperative complication rate decreased significantly from 57% to 15% (*p* = 0.01), Clavien > III complications decreased from 50% to 7% (*p* < 0.01), and hepatic recurrences were also significantly reduced (40% to 5%, *p* = 0.03). Conclusion: Centralization of the surgical management of liver tumors in Switzerland has improved quality of care in our center by significantly reducing postoperative complications and hepatic recurrence.

## 1. Introduction

Liver tumors are rare in children, accounting for 1% of all pediatric tumors. Approximately two-thirds of cases are malignant, with hepatoblastoma (37%), hepatocellular carcinoma (21%), and undifferentiated embryonal sarcoma of the liver (8%) being the most frequent ones; one-third are benign tumors, such as hemangioma, focal nodular hyperplasia, and mesenchymal hamartoma [1]. Although rare, liver tumors are associated with prematurity, chronic hepatitis, Beckwith–Wiedemann syndrome, neurofibromatosis, trisomy 21, and other conditions that may favor their occurrence [2].

Liver tumors are mostly asymptomatic for a long period of time and are often detected during abdominal palpation by the parents or the pediatrician [3]. The differential diagnosis of a hepatic mass is extensive and varies according to age. In newborns and infants, the most common tumor encountered is hemangioma, followed by mesenchymal hamartoma and hepatoblastoma; in children under five years of age, 90% of the malignant tumors are hepatoblastomas, and in those older than five years, hepatocellular carcinoma is most frequently seen [4].

Surgery with complete resection is the gold-standard in the treatment of patients with a hepatic mass. Whether patients receive (neo-)adjuvant chemotherapy depends on the protocol used and the tumor stage of the disease [5]. Liver surgery is complex, requiring specific technical expertise, and is often burdened with complications. Reported morbidity after pediatric liver resections ranges between 15.5% and 69.2%, with a pooled morbidity of 33.9%; surgery-related mortality was reported to be <5% [6].

Centralization for complex surgical procedure is being increasingly recognized to improve the quality of patient care, to optimize the use of resources, and to improve patient outcome [7]. In Switzerland, starting in 2009, the Swiss Conference of Cantonal Health Directors has assigned different specialties to “highly specialized medicine.” Thus, pediatric liver surgery received this status in 2012 and accordingly was centralized to a single, national center, the University Hospitals of Geneva (Swiss pediatric liver center) [7,8,9,10,11]. Before 2012, pediatric liver surgery was performed in seven different centers in Switzerland.

The objective of this study was to analyze the clinical data concerning the surgical management of pediatric patients operated for liver tumors at the Swiss pediatric liver center and to analyze outcomes in the two cohorts before and after centralization. We hypothesized that outcome improved in the second period, the primary outcome being the rate of postoperative complications.

## 2. Materials and Methods

**Figure 1:**
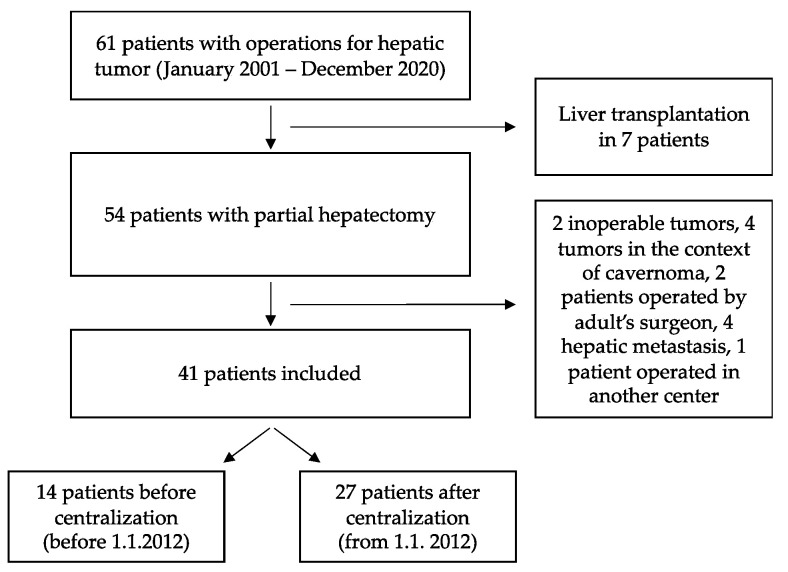
Flow-chart of included and excluded patients.

This is a retrospective monocentric comparative study (historical cohort) based on data from medical records of patients treated for any liver tumor between 1 January 2001 and 31 December 2020 at the University Hospitals of Geneva. During this period, 61 children (0–16 years old) were treated for a benign or malignant primary liver tumor. Patients who had a total hepatectomy and received a liver transplant (*n* = 7), atypical hepatectomy for metastases (*n* = 4), hepatic tumor in the context of a cavernoma (*n* = 4), unresectable tumors (*n* = 2), as well as previous hepatic surgery performed at another center *(n* = 3) were all excluded from further analysis. Finally, 41 patients met the inclusion’s criteria: 14 patients before centralization (January 2001–December 2011) and 27 patients after centralization (January 2012–December 2020) (Figure 1).

We collected epidemiological data (sex, gestational age at birth, birth weight, comorbidities, age at diagnosis), preoperative data (type of tumor, extension, rupture, biopsy, number of neoadjuvant chemotherapy cycles, time from diagnosis to surgery, time from last chemotherapy to surgery, administration of preoperative Granulocyte-Colony Stimulating Factor (GCSF), portal embolization or other preoperative intervention), intraoperative data (weight at surgery, type of resection, R0 margins, duration of surgery, vascular exclusion and its duration), and postoperative data (length of stay, type and number of complications, disease relapses, number of adjuvant chemotherapy cycles, duration of follow-up, and mortality).

The used statistical methods were purely descriptive: the continuous variables were expressed as median with the respective interquartile ranges and/or in percentage. For categorical data, we used the Fischer’s exact test to analyze the different variables of the two study groups; continuous data were compared using the *t*-test. A *p*-value of <0.05 was considered statistically significant.

## 3. Results

Demographic, clinical, and pathological characteristics of the study population and comparisons between the two subgroups are summarized in Table 1.

The two cohorts (before and after centralization) were homogeneous. There were no differences between groups in regards to gender distribution, age at diagnosis, prematurity, birth weight, associated conditions or syndromes (the most frequent being the Beckwith–Wiedemann syndrome), tumor characteristics, and weight at surgery. The only difference between the two groups was that all patients in the recent era had a biopsy before surgery, whereas only 80% had one in the early group.

In both groups, roughly 75% of liver tumors were malignant, the most frequent tumor being hepatoblastoma. Among the benign tumors, the most frequent were mesenchymal hamartoma and focal nodular hyperplasia. In terms of presentation at diagnosis, the majority of malignant tumors were neither ruptured nor metastatic.

Table 2 compares the pre-, peri-, and postoperative data of the two study subgroups. There was no statistically significant difference between the two groups in terms of preoperative GCSF administration, preoperative portal embolization, other abdominal surgery before liver surgery, type of liver resection, and resection margins (R0 resection); all operations were open surgeries. Operating time was reduced by 96 min after centralization (366 min vs. 270 min) but did not reach statistical significance. The number of cases receiving vascular exclusion during hepatectomy was significantly lower after centralization (29% vs. 0%, *p* = 0.01). There was also no statistically significant difference between the two groups for length of stay and 30-day mortality. The number of surgeons performing the liver resections was not different before and after centralization. The number of annually performed hepatectomies increased significantly by three times from one to three per year (*p* < 0.0001) before and after centralization, respectively.

Overall, postoperative complications were significantly reduced after centralization, decreasing from 57% to 15% (*p* = 0.01). Details of complications in the two subgroups are described in Table 3. If only Clavien III complications and higher were considered, significance increased to *p* < 0.01, with an extensive lower rate after centralization (50% versus 7%). When complications were dichotomized into “early” (less than one month after surgery) and “late” (beyond one month after surgery), there was no statistically significant difference between the two subgroups for “early” but a statistically significant difference for “late” complications, which decreased from 50% before to 25% after centralization (*p* = 0.04). Those late complications were exclusively local hepatic recurrences in patients with malignant tumors, which occurred in 40% of patients before centralization and in 5% of patients after centralization (*p* = 0.03).

## 4. Discussion

This study, in the setting of children needing liver surgery, scientifically confirms the intuitive impression that an increased case load after centralization reduces the rate of postoperative complications and hence increases the quality of care.

The median age of patients in our study cohort was two years, and the most frequently encountered malignant tumor was hepatoblastoma, both in accordance with the literature [12,13].

Centralization is a strategy that aims to improve the quality of care and is particularly applied to the most specialized fields of medicine. Indeed, several European countries have felt the need to centralize subspecialties of surgery in a single center [14]. A special edition of the European Journal of Pediatric Surgery addressed this issue in 2017: Durkin et al. [15], Wijnen et al. [16], and Pintér et al. [17] described the motivations and the importance of centralization in the United Kingdom, the Netherlands, and Hungary, respectively, yet unfortunately without offering any statistical results. Only one analytic pediatric study exists from Sweden [18], which studied the mortality rate associated with cardiac surgery before and after centralization: before centralization, the overall 30-day mortality was 9.5%, which decreased to 1.9% after centralization. Interestingly, centralization does seemingly not only improve outcome but can also influence the epidemiological landscape: in the Netherlands, renal tumors have been centralized from 2015 and first results, published in 2022, showed that centralization of care for children with renal tumors led to referral of more than expected cases. Further and very promising, national centralization led to enhanced development of molecular diagnostics and other innovation-based treatments for the future [19]. In adults, studies addressing the effect of centralization are much more numerous and conclusive. For example, Sheetz et al. [20] showed a difference in outcomes after centralization for high-risk oncological surgeries in the United States of America. In particular, the authors demonstrated a reduced rate of complications and death for lung resection, esophagectomy, and pancreatectomy and an absolute reduction in 30-day mortality after pancreatectomy for each 20% increase in the degree of centralization within systems. In Canada, Siemens et al. [21] identified 5574 adult patients who underwent radical cystectomy. The mean annual surgeon volume and hospital volume of radical cystectomy from 1994 to 2008 was a low 4.5 (95% confidence interval (CI) 4.4–4.7) and 12.2 (95% CI 11.8–12.5), respectively. From 2009 to 2013, these volumes increased significantly to 6.8 (95% CI 6.5–7.1) and to 16.4 (95% CI 15.8–16.9), respectively. Over the study period, there was a substantial improvement in cancer-specific survival, and hence, the study clearly showed that there was a real improvement in quality of care through the “passive centralization” of this procedure (there was no active, compulsory centralization). Similar results were presented in a recent systematic review and meta-analysis regarding pancreatic cancer resection: the authors confirmed a clear relationship between patient volume and clinical outcome in this setting. Interestingly, they also showed that more complex surgeries were performed in high-volume centers; however, these more complication-prone surgeries did not worsen outcome, such as mortality, morbidity, failure-to-rescue, and positive resection margin rates [22]. This is also valid for the centralization of highly complex low-volume procedures in upper and lower gastrointestinal surgery. A systematic review and meta-analyses showed that a majority (>90%) of the twelve reviews revealed a lower mortality after complex upper gastrointestinal surgery in high-volume hospitals [23]. The same seems true for colorectal cancer surgery: in a review including 23 articles, high-volume providers had a significantly lower postoperative mortality and better long-term survival for their patients [24]. In short, all these studies and results come with one common single conclusion: centralization favors better patient outcome, in adults as in children.

Although studies have shown that outcome is improved for institutions with “high-volume surgeons,” compared to surgeons performing less frequently certain types of complex surgery [24,25], it is essential to mention that the positive effect of centralization on postoperative complications is certainly not only due to the increased experience of surgeons but also attributable to the entire treating institution. In our study, the streamlined and protocoled multidisciplinary care by the entire team undoubtedly must have contributed to the better outcome. The larger volume of surgeries after centralization allowed for implementation of pre-, peri-, and postoperative protocols, and we feel the entire team was more at ease with these patients and procedures. It has long been shown that standardized protocols improve outcomes for patients after surgical procedures, as demonstrated in 1998 by Bradshaw and Thirlby in the setting of colorectal surgery [26], and also Clark et al. showed the positive effect of protocols in a study analyzing open hepatic surgery in adults [27].

Our study clearly confirms the effect of increased case volume after centralization: in our center, overall and severe postoperative complications were significantly reduced after national centralization. Before centralization, complications occurred in more than one out of two patients and after centralization, in only one of seven patients. There were no major changes of surgical management before and after centralization neither in GCSF administration, in the number of performed embolization procedures before surgery, nor type and quality of resections. This absence of major descriptive differences between the two subgroups reinforces the stated hypothesis: the only significant change in reducing postoperative complications seems to be the increased number of procedures performed by the surgical team and thus their exposure to this complex surgery even more so since surgical devices have very little changed over the last two decades and are not thought to have contributed as drastically to the better results. Of note, in Switzerland, before 2012, hepatectomies were performed by seven different teams, and thus, surgeons were rarely exposed to this type of surgery, as it is seen from our numbers: during the 11 analyzed years before centralization, only 14 cases were operated in our center, whereas during the 9 years after centralization, 27 cases were operated in this same center, increasing exposition by three times and thus increasing expertise. The volume of 27 hepatectomies in the 9 years after centralization reveals the recent national incidence of pediatric liver tumors in Switzerland. This means that before 2012, the three hepatectomies per year in Switzerland were operated in the seven different centers performing this surgery, showing that the case volume in each center ranged from very low 0 to 1 hepatectomies per year, presumably too low to allow for sufficient exposition and consequent expertise. However, and of note, we lack clinical outcome data to underline this conclusion. 

Naturally, we would have expected a decrease in operating time and length of stay after centralization. The decrease of one and a half hours operating time did not reach statistical significance, probably due to small numbers in our cohort. However, this will probably become significant in the future, when more patients will be included in the analysis. As for length of stay, this clearly appears to be a multifactorial variable. Not only do surgical factors but also the fact that patients now come from further afield contribute to a longer hospital stay. This probably contributes to this non-significant difference.

In the early era, forty percent of our studied children with malignant tumors had a local relapse of their tumor, whereas only 5% in the recent cohort, the difference being significant. The collected data show a difference in the number of cycles and type of neo-adjuvant chemotherapy, which can be explained by changes in SIOPEL/PHITT protocols over the years. The reduced number of recurrences might thus be partially attributable to oncological advances with constantly evolving protocols but probably also to improved surgical management, i.e., increased experience of the surgical team due to amplified exposure and thus expertise. Of note, hepatic recurrences occurred at a median of one year after initial hepatectomy, which refutes the explanation that only the shorter follow up of the recent subgroup explains the observed difference.

Our study has several limitations. The retrospective nature cannot guarantee the accuracy of all collected variables. The limited number of cases, even though the cohort “after centralization” includes all Swiss cases of liver tumors, limits the power of the study and therefore the interpretation of the results; yet, results are clearly significant and thus counterbalance the possible lack of power. The form of our study could evoke selection biases because the population before centralization would not be the same as after centralization (we might, for instance, expect an increase in severe cases in the second period); however, our analysis shows that the only parameter that changed between the two eras was the “catchment area.” Indeed, there is no statistically significant difference between the two subgroups neither in terms of epidemiological data nor in terms of pre- and peri-operative descriptive data.

## 5. Conclusions

The increased case load after centralization of the surgical management of liver tumors in Switzerland has significantly improved the results of the management of children undergoing hepatectomy in our center, in particular by significantly reducing postoperative complications. These results represent, to our knowledge, the first pediatric study in the field and are consistent with those found in the adult literature. A study with a larger population would improve the power of the obtained results. Nevertheless, our results clearly strengthen and support the trend for the centralization of highly specialized pediatric surgery.

## Figures and Tables

**Table 1:** Demographic, clinical and pathological characteristics of the study population and the two subgroups. * Beckwith–Wiedemann syndrome (3), neurofibromatosis type 1 (1), prematurity associated comorbidity (2), congenital porto-systemic shunt (1), trisomy 18 (1), umbilical cord cysts (1).

		Total Cohort (*n* = 41)	Before Centralisation (*n* = 14)	After Centralisation (*n* = 27)	*p*-Value
Gender, *n* (%)	Male	24 (59)	7 (50)	17 (63)	0.51
	Female	17 (41)	7 (50)	10 (37)	
Age at diagnosis (months), median (IQR)		24 m (9–77 m)	21 m (7–132 m)	24 m (10–54 m)	0.13
Weight at surgery, median (IQR)		12 kg (9–20 kg)	13 kg (9–30 kg)	12 kg (9–17 kg)	0.20
Birth data		*n* = 22	*n* = 7	*n* = 15	0.63
Term born (38–42 SA), *n* (%)		15 (68)	4 (57)	11 (73)	
Pre term (< 38 SA), *n* (%)		6 (27)	3 (43)	3 (20)	
Late term (> 42 SA), *n* (%)		1 (5)	0 (0)	1 (7)	
Birth weight, median (IQR)		2840 g (2509–3473 g)	2645 g (2635–2898 g)	2840 g (2145–3473 g)	0.99
Comorbidity *	Yes, *n* (%)	9 (22)	4 (29)	5 (19)	0.69
	No, *n* (%)	32 (78)	10 (71)	22 (81)	
Biopsy before surgery	Yes, *n* (%)	38 (93)	11 (79)	27 (100)	0.03
	No, *n* (%)	3 (7)	3 (21)	0 (0)	
Tumor characteristics					0.71
Benign disease, *n* (%)		10 (24)	4 (29)	6 (22)	
Mesenchymal hamartoma		4	2	2	
Focal nodular hyperplasia		3	2	1	
Lipoblastoma		1	0	1	
Hemangioma		1	0	1	
Adenoma		1	0	1	
Malignant disease, *n* (%)		31 (76)	10 (71)	21 (78)	
Hepatoblastoma		27 (87)	7 (70)	20 (95)	
Embryonal sarcoma		4 (23)	3 (30)	1 (5)	
Rupture (R) and/or metastases		*n* = 31	*n* = 10	*n* = 21	
(M) at diagnosis					
R– M–, *n* (%)		19 (61)	6 (60)	13 (62)	
R+ M–, *n* (%)		7 (23)	1 (10)	6 (28)	
R– M+, *n* (%)		4 (13)	2 (20)	2 (10)	
R+M+, *n* (%)		1 (3)	1 (10)	0 (0)	

**Table 2:** Pre-, peri-, and post-operative characteristics of the two subgroups before and after centralization.

		Before Centralization (*n* = 14)	After Centralization (*n* = 27)	*p*-Value
Cycles neoadjuvant chemotherapy		*n* = 10	*n* = 21	
Median (IQR)		2 (0–4)	4 (3–4)	0.76
GCSF administration before surgery, *n* (%)		1 (7)	3 (11)	1.00
Embolization before surgery, *n* (%)		2 (14)	2 (7)	0.60
Prior abdominal surgery before hepatic surgery, *n* (%)		1 (7)	1 (4)	1.00
Type of resection, *n* (%)				
Right lobectomy		3 (21)	7 (26)	1.00
Left lobectomy		2 (14)	6 (22)	0.69
Extended right lobectomy		3 (21)	3 (11)	0.39
Left lateral segmentectomy		2 (14)	5 (19)	1.00
Extended left lobectomy		2 (14)	1 (4)	0.26
Segmentectomy		1 (7)	3 (11)	1.00
Atypical resection		1 (7)	2 (7)	1.00
Surgical margins, *n* (%)	Negative	14 (100)	27 (100)	1.00
	Positive	0 (0)	0 (0)	
Operative time in minutes, median (IQR)		366 (302–496)	270 (219–335)	0.17
Vascular exclusion	Number, *n* (%)	4 (29)	0 (0)	0.01
	Minutes, median (IQR)	71 (55–81)	- (-)	
Days of postoperative stay, median (IQR)		7.0 (7.0–10.0)	8.0 (7.0–9.5)	0.37
Post-operative’s cycles chemotherapy, median (IQR)		*n* = 10	*n* = 21	
		2 (1–3)	2 (2–2)	0.29
Surgical complications, *n* (%)		8 (57)	4 (15)	0.01
Duration of follow-up in months, median (IQR)		5 (1–23)	47 (12–60)	0.15
30-day mortality, *n* (%)		0 (0)	0 (0)	

**Table 3:** Surgical postoperative complications and hepatic relapses before and after centralization.

	Before Centralization (*n* = 14)	After Centralization (*n* = 27)	*p*-Value
All complications, *n* (%)	8 (57)	4 (15)	0.01
Complications Clavien III and higher, *n* (%)	7 (50)	2 (7)	<0.00
Early (<1 month), *n* (%)	4 (50)	3 (75)	0.21
Respiratory, *n* (Clavien classification)	2 (IIIb)	2 (I)	
Infectious, *n* (Clavien classification)	1 (II)	0 (-)	
Biliary, *n* (Clavien classification)	1 (IIIb)	0 (-)	
Intestinal, *n* (Clavien classification)	0 (-)	1 (IIIb)	
Late (>1 month), *n* (%)	4 (50)	1 (25)	0.04
Surgical complications	0	0	
Malignant tumors	*n* = 10	*n* = 21	0.03
Hepatic relapses, *n* (%)	4 (40)	1 (5)	

## Data Availability

The data presented in this study are available on request from the corresponding author.
